# Supplementary material for: Are carbon emissions trading and green financial instruments synergistic? -Comprehensive quantitative research based on content analysis
Source: PLoS One. 2024 Mar 7;19(3):e0298601. doi: 10.1371/journal.pone.0298601 (PMC10919601; doi:10.1371/journal.pone.0298601)
Supplement: S1 Data — (ZIP) [file pone.0298601.s001.zip › Data/地级市Z_F工作报告.zip!/╔╜╢1⁄2/▒⌡╓▌╩╨/└┤╫╘╣1⁄2╓┌║┼í╛┬φ┐╦╩2╛▌═°í┐_2009.docx]

各位代表:

　　现在,我代表市人民政府向大会作政府工作报告,请予审议,并请市政协委员和其他列席会议的同志提出意见。

　　一、2008年工作回顾

　　2008年是极不平凡的一年。世界金融危机日益加深,国内经济发展急剧趋缓,我市经济运行遇到前所未有的困难。面对复杂多变的严峻形势,市政府在市委的坚强领导下,在历届领导班子打下的良好基础上,紧紧团结依靠全市人民,深入贯彻落实科学发展观,解放思想,把握规律,攻坚克难,科学实干,全市经济社会保持了平稳健康发展的良好态势。

　　(一)综合经济实力迈上新台阶。预计全市实现生产总值1230亿元,增长12.7%;城镇居民人均可支配收入15960元,农村居民人均纯收入5700元,分别增长14.9%和14.3%。三次产业比例调整为9.9:61.4:28.7。完成地方财政收入70.37亿元,增长18.5%,财政支出首次突破100亿元大关。抢抓国家扩大内需、加大投资的机遇,争取资金高于全省平均水平。

　　农业基础地位不断加强。预计全市实现农业总产值240亿元,增长5%。按照“粮丰林茂、北国江南”的目标定位,深入开展林水会战,新增合格造林面积33.9万亩,比上年增加26.5%;新增节水灌溉面积10.2万亩;新增蓄水能力1980万方,全市一次性总蓄水能力达到7.7亿方。黄河标准化堤防建设第二阶段工程竣工。解决了29.1万农村居民饮水问题,全市农村自来水普及率达到98%。继续实施“村村通公路”工程,新建、改造农村公路1396公里。规模以上农业龙头企业达到470家。启动建设“滨州超有机农产品生产示范基地”。落实粮食直补、良种和农资等惠农补贴3.6亿元。粮食总产达到277万吨,增长5%。重大动物疫病免疫密度达到100%。完成270个社会主义新农村示范村达标建设任务。

　　“工业兴市”扎实推进。大力实施“11426”计划,以结构调整为主线,以园区建设为载体,积极壮大骨干企业,着力提升企业核心竞争力。预计规模以上工业完成增加值660.6亿元,增长13%。预计工业对经济增长的贡献率达到52%。规模以上工业企业达到1358家,主营业务收入过100亿元企业达到5家。新增中国驰名商标2件、山东名牌产品15个、著名商标9件。生态纺织品质检中心滨州实验室、检验检疫棉花实验室获得国家认证。

　　加快经济发展方式转变。预计全年高新技术产业产值达到380亿元,增长26%,占规模以上工业总产值比重达到13.7%。高新技术产业开发区筹建工作开始启动。新增省级企业技术中心3家。创建“中国优秀旅游城市”工作通过国家验收。加快企业二、三产业剥离,财税结构不断优化。预计服务业增加值354亿元,增长18%,占生产总值的比重达到28.8%。金融机构本外币存、贷款余额分别达到700亿元和669.4亿元,比年初增长23.7%和21.4%。第七届银企合作促进会签约资金820.5亿元,增长15.6%。民营经济实缴税金82.8亿元,增长15.5%。县域经济实力增强,帮扶阳信、惠民工作效果明显。预计全市万元生产总值能耗降低5.8%,二氧化硫和化学需氧量减排分别完成“十一五”总任务的60%以上,实现了全年节能减排目标。

　　对外开放取得新成绩。预计实现进出口贸易总额46.2亿美元,增长37.5%;其中出口23.2亿美元,增长23.2%。实际利用外资3亿美元,增长19.4%。1家企业在欧洲成功上市,全市境外上市企业达到5家,融资70多亿元。与日本纪之川市缔结友好城市关系。滨州经济开发区、邹平经济开发区荣获“山东省对外开放先进园区”称号,全市8个省级开发区辐射带动作用日益增强。基础设施建设进展良好。预计全市规模以上固定资产投资完成578.7亿元,增长21%。滨州大港建设实现重大突破,2个3万吨级散杂货泊位岸线获得交通运输部批复,项目已经省发改委立项,引堤主体工程基本完工。万吨级深水航道及防波堤、套尔河3000吨级航道及防波堤工程可行性研究报告通过省交通厅审查。沾化一级渔港开工建设,内河港区建设有序进行。北海新区开发顺利起步,各项规划编制扎实推进。圆满完成省政府下达的耕地保护责任目标。滨州贝壳堤岛与湿地国家级自然保护区调整方案已经环保部组织专家论证通过。滨德高速公路、黄大铁路(黄河特大桥)开工建设。滨港铁路一期工程已具备开通条件。疏港公路一期工程建设任务按期完成。205国道、永莘路等八条干线公路重点路段改造开工建设。滨州客运总站主体基本竣工。国华瑞丰风电、鲁北电力等一批能源项目建设顺利进行。

　　城市建设展现新面貌。创建省级园林城市工作步伐加快,城区绿化覆盖率达到36.8%,人均公共绿地面积达到9.9平方米。城区基础设施建设及旧城改造工程圆满完成,功能日趋完善,承载能力显著增强。大力开展城乡环境综合整治活动,积极实施“管理、执法、服务”三位一体城市精细化管理模式,市容市貌明显改观。张肖堂干渠和滨南支沟治理工程胜利竣工。全市邮电通信体制改革顺利完成。

　　(二)各项社会事业全面进步。科教建设继续加强。科技投入力度加大,预计全市科技支出1.2亿元。素质教育全面推进,教育质量明显提高,高考成绩再创新高。全部免除农村义务教育阶段学生教科书费。农村义务教育阶段寄宿学生生活补助标准、农村中小学生均公用经费定额得到提高。全市62处危改学校项目完成投资1.1亿元。城区义务教育阶段学校建设步伐加快。高等教育、职业教育水平进一步提高。

　　文化、体育、卫生、人口和计生等工作统筹发展。博兴董永传说、博兴吕剧、滨州剪纸、沾化渔鼓戏成功入选第二批国家级非物质文化遗产名录。十一届全运会场馆建设进度加快,竞技体育、群众体育进入全省前列。精神文明建设迈上新台阶,被授予“省级创建文明城市工作先进城市”称号。成功举办家纺文化节、市第六届体育节、孙子文化旅游节等重大节会赛事活动。城市社区卫生服务覆盖率达到80%;乡镇卫生院建设改造任务全面完成,管理体制改革稳步推进;农村社区卫生服务站建设和卫生室改造快速展开。手足口病得到有效控制。我市被授予“全国红十字服务示范市”荣誉称号。人口和计生工作完成年度责任目标。巩固双拥共建成果,国防教育、人民武装工作得到加强。“平安滨州”建设成效明显。严格落实“一岗双责”责任制,加强社会治安综合治理,“北京奥运护城河工程”取得重大胜利,社会和谐稳定局面进一步巩固。食品药品监管力度加大,打击制售假冒伪劣商品取得新成效。深入开展“隐患治理年”活动,落实责任,依法监管,全市安全生产形势总体稳定。民族宗教、妇女儿童、广播电视、新闻出版、外事侨务、统计、物价、残疾人事业、对台、老龄、史志、地名、地震、气象、海事、人防、油区管理等各项工作都有了新的进步。

　　(三)保障和改善民生成效显著。社会保障体系逐步完善。城乡一体化就业体系初步形成,新增城镇就业3.7万人,其中失业人员再就业1.2万人,安置困难群体再就业4232人,新增农村劳动力转移就业6.3万人。城镇登记失业率3.07%。社会保险覆盖面进一步扩大。城镇居民基本医疗保险试点工作参保人员达到19.2万人。贫困学生救助体系逐步完善。居民消费价格同比上涨4.8%,低于全省平均水平。新农合运行机制不断规范完善,参合人数达到288.7万人,参合率95.8%。提高了低保标准和补助水平,城乡低保对象实现应保尽保。积极做好高龄特困救助工作。五保供养政策得到全面落实,78处敬老院完成建设任务,集中供养率达到72%。经济适用房竣工面积7.1万平方米,完成年初计划的100.6%。廉租住房保障范围扩大到城市低收入住房困难家庭。

　　支援抗震救灾和对口援建工作取得阶段性成果。全市动员,众志成城,共接收捐赠款和物资折款1.4亿元,组织消防、武警、卫生等队伍奔赴四川地震灾区抢险救灾。科学编制禹里乡恢复重建规划,过渡性学校建设全面完成。

　　(四)政府自身建设进一步加强。坚持依法行政,认真执行人大及其常委会的决议、决定,及时向政协通报情况,认真办理人大代表建议和政协委员提案,切实密切与各民主党派、工商联、无党派人士和工会、共青团、妇联等人民团体的联系。深入开展新一轮解放思想大讨论活动,推动工作作风转变,服务水平明显提升。深化行政审批制度改革,减少行政审批事项114项,符合条件的审批事项按时办结率达到100%。强化政务公开,全面建立政府信息公开机制,阳光政府建设迈出新步伐。处理市长公开电话6.2万件、群众来信1141件。政府应急管理工作全面推进,处置突发事件的能力不断提高。抓好招投标和稽查工作,推行部门预算和政府集中采购等制度,强化监察和审计监督,扎实开展商业贿赂专项整治,严肃查处了一批违法违纪案件,廉政建设得到加强。严格实施行政问责,规范权力运行,发展环境进一步优化。

　　各位代表!一年来,在各种困难和挑战面前,我们取得这样的发展成绩,来之不易。这是省委、省政府和市委正确领导、亲切关怀的结果,也是全市人民锐意进取、实干创业的结果。在此,我代表市人民政府向全市各族人民,向各民主党派、工商联、各人民团体和社会各界人士,向驻滨人民解放军、武警官兵和中央、省驻滨单位,向所有关心、支持滨州发展的海内外朋友,表示衷心的感谢和崇高的敬意!

　　总结一年来的工作,我们也清醒地认识到,经济社会发展和政府工作中还存在许多问题。

　　一是贯彻落实科学发展观的自觉性还不够高。学习不够扎实、领会不够全面、理解不够深入,缺乏战略思维、辩证思维、系统思维和创新思维的能力。

　　二是经济结构不够合理的问题还比较突出。传统产业比重偏高,粗放型发展方式尚未明显改变,发展质量和效益不高,自主创新能力不强,服务业发展相对滞后,南北发展差距没有明显改观。

　　三是解决民生问题有待进一步加强。群众就业、上学、看病、住房、养老等方面还存在一些突出问题。社会保障能力需进一步加强,就业形势十分严峻,有的城乡群众生活还比较困难。

　　四是政府管理职能、运作机制还需进一步完善。政府职能的越位、缺位、错位现象依然存在,群众对干部作风和政府工作效率还不够满意,极少数干部的不良行为影响很坏。

　　这些问题如不及时解决,我们就无法应对国际国内形势带来的严峻挑战,就无法实现我市经济社会又好又快发展,就难以肩负起带领全市人民建设新滨州的崇高使命。我们时刻要保持清醒的头脑,深刻认识存在的问题和不足,深刻认识面临的困难和挑战,深刻认识我市发展的阶段性特征,深刻认识我市在全国、全省发展大局中的方位和水平,不为过去的成绩而满足,进一步解放思想,更新观念,切实增强忧患意识、责任意识、大局意识、赶超意识,树立更高的目标追求,永不自满、永不懈怠地做好各项工作,决不辜负全市人民的期望和重托!

　　二、20 09年奋斗目标和工作重点

　　2009年是新中国成立60周年,是推进“十一五”规划顺利实施的关键一年。当前,国际金融危机仍在扩散和蔓延,对全球经济的冲击和造成的损失将进一步扩大,对我市经济发展影响的时间和程度存在很大的不确定性。我市经济运行中的一些困难和问题难以预料,我们将面临严峻的考验和挑战,保增长将是今年经济工作的首要任务。2009年也是滨州深入学习实践科学发展观,抢抓机遇、实现跨越发展的关键一年。面对挑战,我们更要充满信心,理性看待挑战与机遇,在困境中攻坚克难,在挑战中抢占先机。近一段时期以来,中央和省果断实施了一系列扩大内需、保持增长的政策措施,省委、省政府出台了34条开发建设黄河三角洲的支持政策。经过改革开放30年来的快速发展,特别是近年来的发展,我市产业体系和一批新的经济增长点正在形成,后发优势日益凸显,骨干企业有信心、有条件渡过暂时的困难。只要我们坚定不移地贯彻发展是硬道理的战略思想,坚定必胜信念,完全有条件变压力为动力、化挑战为机遇,就一定能够克服前进道路上的艰难险阻,夺取全市经济社会发展的新胜利!

　　今年政府工作的指导思想是:全面贯彻党的十七大、十七届三中全会和中央、全省经济工作会议精神,以邓小平理论和“三个代表”重要思想为指导,深入落实科学发展观,坚持“30字”工作方针和“16字”总体要求,突出投入拉动、结构调整、改革开放、城乡统筹、基础设施建设、民生改善、文化繁荣、稳定和谐“八个重点”,强化科学实干、优化环境、创新机制“三大保障”,进一步解放思想,坚定信心,积极作为,科学务实,精心打造“四环五海、生态滨州”、“粮丰林茂、北国江南”两个品牌,奋力建设富裕、文明、和谐的新滨州。

　　今年国民经济和社会发展的主要预期目标是:生产总值增长12%;规模以上固定资产投资增长22%;地方财政收入增长10%;规模以上工业增加值增长13%;社会消费品零售总额增长16.5%;外贸进出口总值增长8%;城镇居民人均可支配收入、农村居民人均纯收入分别增长8%和9%;居民消费价格涨幅控制在4%以内;城镇居民登记失业率控制在4.6%以内;人口自然增长率控制在5.5‰以内;万元地区生产总值能耗下降5.83%;二氧化硫和化学需氧量完成“十一五”减排任务的90%。

　　各位代表,在世界经济减速、外部环境复杂多变的形势下,提出生产总值增长12%、地方财政收入增长10%的目标,主要是着眼于“保增长”的要求,这有利于稳定社会预期、增强发展信心,对于扩大城乡就业、加快发展社会事业和缓解突出矛盾十分必要。今年要全力抓好十项重点工作。

　　(一)保持经济平稳较快增长。越是经济低谷,越有突破机会。认真落实国家和省一系列政策措施,把扩大内需作为促进经济增长的重要途径。

　　保持投资较快增长。把狠抓项目、扩大投资作为保增长的主攻方向和着力点,抓好大项目建设,加快推进开工项目,策划实施一批重大项目,建设一批、储备一批、规划一批,努力保持投资的持续拉动。加大投资力度,优化投资结构,加快培育新的经济增长点,增强可持续发展能力。在充分发挥政府投资拉动作用的同时,积极引导和鼓励社会投资。严控“两高一资”项目和产能过剩投资,坚决防止低水平重复建设和盲目扩张,确保投资质量,提高投资效益。进一步扩大消费。增强消费对经济增长的促进作用,采取多种措施,努力提高农民和城市低收入者的收入水平,提高居民消费能力。加强农村流通设施建设,继续搞好“万村千乡”市场工程,加快农产品经营服务体系建设,扩大农村消费。加大保障性住房建设力度,鼓励普通商品房消费,落实二套房政策,促进房地产市场健康发展。

　　准确把握国家宏观调控政策。抢抓机遇,搞好规划,进一步增强工作针对性和灵活性,主动与国家、省有关部门进行沟通衔接,积极争取更多重点项目和资金支持,为今后更长时间的发展奠定基础。

　　(二)大力推进农村改革发展。“三农”问题是事关全局的重大问题。扩大内需的最大潜力在农村。认真贯彻党的十七届三中全会精神,推进农村改革发展,是促进农村经济社会全面进步的迫切要求,是农业增产、农民增收、农村繁荣的必由之路。

　　加快建设现代农业。大力发展农牧渔果等特色产业,突出发展农业龙头企业,推动各类农产品区域化、规模化、标准化生产基地建设,加快发展现代循环农业产业化。抓好“超有机农产品生产示范基地”建设。建立完善农产品质量安全体系,实行最严格的农产品质量安全、重大动植物疫病防控责任制。

　　切实加强农业基础建设。紧紧围绕“粮丰林茂、北国江南”的目标定位,始终抓住林水会战和粮食生产不放松。按照规划,有序推进农田水利基本建设和植树造林,加快实施农业综合开发。抓好六个大型引黄灌区续建配套与节水改造、黄河标准化堤防第三阶段工程建设。实行粮食生产县(区)行政首长负责制,加大中低产田改造和高产创建力度,稳定粮食播种面积,争取粮食生产再获丰收。大力发展农村二三产业。重点扶持发展一批中小企业,拉长产业链条,促进产品转化增值。积极发展各类农民专业协会和经济合作组织,提高农民组织化程度,尽快构建联结关系紧密、带动能力强的农业产业化经营体系。加大农村劳动力培训力度,大力支持就业创业,切实保护农民工合法权益。落实各项惠农强农政策,做好农民负担监督管理工作,促进农民持续增收。

　　进一步深化农村改革。稳定和完善农村基本经营制度,积极探索促进农民土地承包经营权流转的新形式。进一步规范完善林权制度改革、小型水利工程产权制度改革。

　　加快发展农村公共事业。扩大公共财政覆盖农村范围,强化农村社会管理,建设社会主义新农村示范村330个。

　　(三)全力抓好“工业兴市”。工业是推动我市经济增长的主要动力。有效扭转经济增速下滑趋势,必须坚定不移地实施“工业兴市”战略,推进工业振兴,坚持走新型工业化道路,强力推进技术进步,促进工业由大变强。

　　着力提升企业竞争力。继续实施“11426”计划,膨胀壮大骨干企业,拓宽延伸工业产业链。积极对接国家十个重点产业调整和振兴规划,把扩大内需、振兴产业和科技支撑结合起来,重点抓好纺织、化工、装备制造、生物医药、新材料、新能源、电子信息等重点产业和重点项目,鼓励企业兼并重组、强强联合,促进产业集聚和产业集群发展。

　　加大企业技术改造力度。充分用好增值税转型、进口设备免税等优惠政策,努力增加技术改造投入,力争全年技术改造投入增长30%以上。着力改造提升纺织、化工、装备制造等传统产业,提高产品档次和附加值,提升产业水平。深入实施品牌战略,积极参加国家、国际标准制定和使用活动,提高产业核心竞争力。加强企业高技能人才队伍建设,培养技师、高级技师400人。

　　拉动中小企业发展。全力实施中小企业培育计划、成长计划、特色产业提升计划和技术创新计划。加强创业示范平台建设,鼓励中小企业与大企业协作配套,促进中小企业转型升级。积极推进中小企业融资担保、创业辅导、信用评价等服务体系建设,继续抓好小额贷款公司试点,缓解中小企业融资难问题。

　　开展支持帮扶企业活动。强化对企业的预警、监测和调度,引导企业加强财务、质量、安全管理,千方百计为企业做好各项服务。简政放权,减轻企业负担,向企业收取的各项规费能减则减,能免则免,与企业共渡难关。积极开拓国际、国内市场,巩固传统市场,开辟新兴市场,抢占市场份额,推动企业营销创新,确保工业经济平稳健康发展。

　　(四)加快重大基础设施建设。抢抓省委、省政府实施“一体两翼”区域发展战略、开发建设黄河三角洲高效生态经济区的机遇,扎实开展“基础设施建设年”活动。

　　进一步完善规划编制。坚持规划先行、环保优先,编制滨州市空间发展战略规划,完善市、县城乡总体规划,完成县域村镇体系规划及新农村示范村建设规划编制。

　　全力抓好十大基础设施工程建设。滨德高速公路完成部分路基土方和桥涵工程;疏港公路二期工程力争开工建设;套尔河大桥主桥、孙武黄河大桥开工建设;滨州大港完成引堤建设,开工建设2个3万吨级通用散杂货码头及防波堤工程、6个5000吨级通用码头工程、套尔河3000吨级航道及防波堤工程;加快推进滨港铁路一期代管运营,做好二期工程开工准备;抓好黄大铁路、德龙烟铁路开工建设工作;积极做好环渤海一级路、渤海大桥等项目前期工作。另外,滨州客运总站“十一”实现试运营。积极推进黄骅至滨州500千伏电网工程。加快长深高速与东青高速连接线前期工作。继续完善防潮堤建设。

　　加快北海新区规划建设步伐。推进基础设施建设,落实各项优惠扶持政策,尽快打造推动滨州发展的重要增长极。

　　完善提高滨州新城区,突破老城区。实施80项城区基础设施建设、改造工程,加快城中村和棚户区改造步伐。积极争创省级园林城市。加强城市精细化管理,抓好迎全运城乡环境综合整治集中行动,提高城市管理水平和服务效率。

　　(五)积极推动经济结构调整。当前,我们面临着保增长的繁重任务,但加快发展方式转变、推动经济结构战略性调整的大方向不能动摇。必须充分运用国际金融危机提供的倒逼机制,加快构建滨州现代产业体系,形成未来发展的新优势。

　　大力发展高新技术产业。深入实施“科技强市”战略,进一步提高自主创新能力,积极扶持装备制造业、生物医药、海洋化工等高新技术产业。发挥企业科技主体作用,推进产学研联合,加强企业技术中心等载体建设,全面发挥人才资源在自主创新中的重要作用,培养和引进高层次创新型人才,加快高新技术产业开发区筹建步伐,积极建设创新型城市。加快发展现代服务业。完善服务业发展规划,抓好体制机制创新和扶持政策的落实,全力培育壮大“三大载体”,着力提升金融保险、现代物流、文化旅游、信息服务等现代服务业发展水平,做大做强一批大型服务企业集团。全面推进旅游强县、强镇、强村建设,大力发展红色、生态、民俗、工业、农业旅游,进一步提高旅游业发展水平。

　　促进县域经济协调发展。强化政策支持,促进企业向工业园区集中、人口向城镇集中,加速形成科学发展、各具特色的现代县域经济。相对发达县区要率先发展,加快结构调整优化力度,增创发展新优势。欠发达县区要坚持从自身实际出发,充分借鉴发达地区的先进经验和教训,大力发展优势特色产业。加大帮扶阳信、惠民力度,努力缩小南北差距,促进县域经济协调、快速发展。

　　(六)进一步深化改革开放。把深化改革、扩大开放作为保增长的强大动力,用创新的思维、改革的办法,解决前进中的困难和问题,进一步激发经济发展的活力。

　　加大重点领域和关键环节的改革力度。加快推进行政管理体制改革,进一步转变职能,加强效能建设,提高政府经济管理和公共服务的水平。积极推进财税体制改革,按照国家统一部署,全面实施增值税转型改革,全面清理行政性收费和政府性基金,全面推进部门预算改革和“乡财县管”,不断深化非税收入管理、政府采购等改革,扎实开展财政资金管理效益年活动。继续调整优化财政收入结构,适时调整支出结构。落实燃油税费改革,认真做好相关配套工作。深化国有企业改革,继续规范优化国有企业股权结构,依法完善公司法人治理结构。提高招商引资实效。突出产业招商、专业招商,重点吸纳一批高精尖项目、产业链系列配套项目。实行更加优惠的招商引资政策,切实做到公开、公正、公平、透明,无论是外来投资者,还是本地投资者,一律享有同等优惠政策。积极培植上市后备资源,加大企业上市工作力度。认真落实适度宽松的货币政策,优化金融环境,改善金融服务,加大金融支持经济力度。组织开展好第八届银企合作促进活动,抓好已签约资金的落实到位。做好企业债券发行、创业投资机构建设,积极探索发行城市建设债券。

　　切实转变外经贸发展方式。培育发展优势出口产业,全面提升出口产业竞争力。大力拓展对外承包劳务,加大境外资源性产品的进口,推动我市纺织、印染、服装、轻工企业到境外经贸园区集聚发展。用足用活上级政策,切实解放思想,放权搞活,进一步优化发展环境,全力支持滨州经济开发区发展,提升其他省级开发区承载能力。

　　(七)强化资源节约和环境保护。今年是完成“十一五”节能减排目标的关键之年,在当前严峻的经济形势下,节能减排措施不能弱化,工作不能放松,建设生态市的目标不能动摇。

　　深入实施“节能减排全民行动”。积极开展循环经济园区和企业创建活动,大力推行清洁生产,进一步淘汰落后产能。严格执行国家产业政策,严把新上项目技术、能耗、环境“三关”,严格环评和“三同时”制度,严控高耗能、高污染行业增长,推动新上项目进区入园,节约、集约发展。

　　健全完善节能减排责任制。加大节能减排执法力度,严格考核监管,确保如期完成“十一五”节能减排目标。抓好重点行业和56户重点用能企业、61户省控重点污染企业的监管工作,推动8家城市污水处理厂满负荷运营,确保达标排放。严厉查处环保违法违规案件,集中解决市辖小清河流域、海河流域水质达标问题。强化能源统计、节能标准化、能源计量等基础性工作。

　　继续推进生态市建设。积极创建国家环保模范城市,优化城乡人居环境。严格自然生态、耕地和基本农田保护,科学编制新一轮土地利用规划,盘活存量土地,提高土地和海域集约利用水平。

　　(八)全面推进社会建设和谐发展。大力发展社会事业,进一步完善社会管理,维护社会稳定,努力提高人民群众的生活质量和水平,为科学发展、加快发展提供和谐的社会环境。

　　优先发展教育事业。全面实施素质教育,推进教育改革创新,完善义务教育经费保障机制。规范发展学前教育,进一步提高义务教育和普通高中教育办学水平,大力发展职业教育,加快高等教育改革发展。

　　大力推进文化繁荣。做大做强黄河文化、孙子文化等滨州特色文化品牌。完成市档案馆、党校等在建工程,全面启动市老干部活动中心、博物馆、艺术馆、图书馆建设。80%的乡镇和村建成综合文化站、文化大院,50%的村覆盖建有农家书屋。深化文化体制改革,推动文化创新,鼓励发展创意、动漫产业,促进文化产业快速发展。积极开展群众性文化活动,加强社区文化、企业文化、村镇文化、校园文化建设,用先进文化占领城乡基层文化阵地。巩固文明城市创建成果,全面提升社会文明程度。按期完成十一运会体育场馆建设任务,确保顺利启用。全力参加全运会,承办好柔道、武术两个项目,积极备战省运会。

　　提高医疗卫生服务水平。开工建设市公共卫生中心。加快农村卫生室改建步伐,推行家庭医生式保健服务。年内乡村卫生一体化管理率、新农合参合率、城市社区卫生覆盖率分别达到100%、98%和100%。全面做好人口和计生工作,圆满完成省下达的任务指标。

　　深入推进“平安滨州”建设。狠抓社会治安综合治理,严厉打击各类违法犯罪活动。认真落实“五五”普法规划,推进依法治市进程。强化矛盾纠纷排查调处,切实做好法律援助工作,促进社会公平正义。深化接访制度改革,开展社情民意调查,拓宽群众诉求渠道。进一步完善城市社区工作机制,建成200个农村社区。严格落实安全生产责任制,深入排查整改安全隐患,坚决杜绝重特大安全事故发生。加强市场监管,确保食品药品、医疗、农资等质量安全。建立完善双拥工作和民兵预备役建设长效机制。高度重视民族宗教工作,积极做好“扫黄打非”、油区、人防、防震减灾、慈善、红十字会等工作。认真做好对口支援四川禹里乡灾后恢复重建工作。

　　(九)着力保障和改善民生。改善民生不仅是构建和谐社会的必然要求,也是扩大内需、促进经济平稳较快增长的重要举措。坚持以人为本,把保障和改善民生作为深入学习实践科学发展观活动的重要内容,全力办好十大“民生工程”。

　　一是就业再就业工程。鼓励创业带动就业,新增城镇就业3万人,新增农村劳动力转移5万人。

　　二是弱势群体基本生活保障工程。落实城乡低保新标准,提高社会救助水平。全部完成敬老院后续建设任务,巩固提高农村五保集中供养率。

　　三是社会保险扩面工程。进一步扩大养老、医疗、失业、工伤、生育保险覆盖面。城镇居民基本医疗保险实现全覆盖。推进新型农村养老保险制度改革。

　　四是社会福利设施建设工程。全面完成渤海革命老区纪念园、儿童福利院建设并投入使用,流浪儿童救助保护中心年内开工建设。

　　五是住房保障工程。建设经济适用住房20万平方米、廉租住房4万平方米。

　　六是农村基础设施建设完善工程。改造、新建农村公路1400公里;改造河道闸危桥57座、农村公路危桥36座;解决和改善农村居民20.75万人饮水安全问题;建设农村沼气池5000个。

　　七是农村中小学改造工程。新建改建校舍14万平方米,实施农村中小学“两热一暖一改”工程。

　　八是困难学生资助工程。落实好普通高校、中职学校和普通高中政府助学金政策,做好全市家庭困难学生资助工作。确保进城务工子女就近接受义务教育。

　　九是全民健康工程。完善基本医疗卫生制度,实现15分钟医疗救治服务圈,确保广大人民群众病有所医。

　　十是老龄、残疾人救助工程。帮助500名残疾人实现就业,帮扶500户贫困残疾人脱贫,为1万名残疾人搞好康复服务。落实90-99岁无离退休金老年人养老补贴制度,完善80岁以上农村老年人免费参加新农合机制。

　　(十)切实加强政府自身建设。面对繁重而艰巨的工作任务,顺利实现今年的各项目标,必须进一步创新政府管理体制和运行机制,确保政令畅通,不断提高领导科学发展的能力和水平。

　　创新管理服务方式。科学、合理界定部门设置,理顺部门职责关系,明确政府职能定位,规范行政权力,避免政府集决策、管理、经营于一身的现象,市场能办的事情交给市场,企业能做的事情还给企业。运用市场机制,支持和鼓励社会民间组织发展,充分发挥他们自我管理、履行义务、奉献社会、志愿服务的作用,政府的职能向更加关注社会管理、公共服务转变。

　　进一步优化发展环境。扎实开展“发展环境优化年”活动,创新政府部门用人机制,坚持能进能出,严格控制机构编制。进一步加快电子政务建设应用,深化政府信息公开工作。继续深化行政审批制度改革,加强网上审批服务和电子监察系统建设,进一步规范行政审批事项,降低行政运行成本。

　　严格落实行政问责制。实施工作目标责任制,严格考核奖惩和监督,健全以行政首长为重点的行政问责制度,明确问责范围,对群众反映强烈的不作为、慢作为、乱作为等行为,加大责任追究力度,切实提高政府公信力。

　　严格依法行政。全面贯彻落实国务院《关于加强市县政府依法行政的决定》,强化行政执法责任制,细化量化行政裁量权。推进科学、民主决策,发挥专家咨询的作用,广泛听取各方面的决策建议。加强监察、审计等行政监督,自觉接受人大及其常委会的依法监督、政协民主监督,接受新闻媒体和社会群众的监督,主动听取各民主党派、工商联、无党派人士和各人民团体的意见。强化反腐倡廉制度建设,严格执行廉洁自律各项规定,切实做到既勤政又廉政,既干事又干净,树立政府为民、务实、清廉的良好形象!

　　各位代表!“长风破浪会有时,直挂云帆济沧海。”站在新的历史起点上,攻坚克难,共渡难关,抓住机遇,加快发展,是滨州人民的期望,是我们的神圣职责和崇高使命。让我们更加紧密地团结在以胡锦涛同志为总书记的党中央周围,在市委的坚强领导下,团结带领全市人民,解放思想,开拓创新,奋力拼搏,为建设富裕、文明、和谐的新滨州做出新的更大贡献,以优异成绩迎接建国六十周年!
